# Intraperitoneal tuberculous abscess mimicking a para-gastric mass: A case report and literature review

**DOI:** 10.1097/MD.0000000000046555

**Published:** 2025-12-19

**Authors:** Wenting Li, Zhikai Zhao, Wei Sha

**Affiliations:** a Department of Tuberculosis, Shanghai Pulmonary Hospital, Tongji University, Shanghai, People’s Republic of China; b Department of Pathology, Shanghai Pulmonary Hospital, Tongji University, Shanghai, People’s Republic of China.

**Keywords:** abdominal abscess, case reports, diagnostic errors, Mycobacterium tuberculosis, peritoneum, tomography, X-ray computed

## Abstract

**Rationale:**

Early diagnosis of abdominal tuberculosis is challenging due to its diverse manifestations. We report a rare case of an intraperitoneal tuberculous abscess that mimicked a tumor, aiming to raise clinical vigilance against abdominal tuberculosis with atypical manifestations.

**Patient concerns:**

A 50-year-old man presented with a 1-month history of abdominal pain.

**Diagnoses:**

Computed tomography imaging demonstrated a multiseptated, peripherally enhancing hypodense mass abutting the gastric greater curvature, initially suspected to be a gastrointestinal stromal tumor. Exploratory laparotomy uncovered a pus-filled mass attached to the gastric fundus. Histopathological examination of the biopsy specimens showed necrotizing granulomas. Polymerase chain reaction assays detected *Mycobacterium tuberculosis* DNA in the specimens. Thus, the diagnosis was determined as abdominal tuberculosis complicating an intraperitoneal tuberculous abscess.

**Interventions:**

The patient was treated with antituberculosis therapy after diagnosis.

**Outcomes:**

The patient reported stable clinical status with no adverse effects during follow-ups.

**Lessons:**

Tuberculosis should be included in the differential diagnosis of intraperitoneal masses to avoid misdiagnosis and unnecessary interventions, particularly in high-TB-burden countries. Diagnosis is based on a multimodal diagnostic approach consisting of clinical manifestations, radiographic, histopathological and microbiological evidence.

## 1. Introduction

Tuberculosis (TB) remains a major global health concern. TB typically affects the lungs but can also affect other sites. According to the World Health Organization’s Global Tuberculosis Report 2024,^[1]^ a record 8.2 million people were newly diagnosed with TB globally in 2023, of which approximately 6.9 million were pulmonary TB cases, indicating that extrapulmonary tuberculosis (EPTB) accounted for an estimated 16% of all notified cases.

Abdominal TB is a common form of EPTB. It can involve various abdominal sites, including the gastrointestinal tract, peritoneum, lymph nodes, and solid organs.^[2]^ The epidemiology of abdominal TB exhibits significant geographical variation. In India, the country with the highest burden, EPTB accounts for 20% of all TB cases, with abdominal TB comprising 12.8% of these.^[3]^ In Pakistan, which bears the fifth-highest global TB burden, abdominal TB constitutes 21% of all EPTB cases.^[4]^ In low-incidence areas like Europe, abdominal TB accounts for only 3% of all EPTB cases.^[5]^

Despite advancements in diagnostic imaging, the diagnosis of abdominal TB remains challenging due to its varied clinical manifestations and radiological features that often mimic malignancy or other inflammatory diseases.^[6,7]^ Consequently, the early and accurate identification of abdominal TB, particularly in its atypical forms, is critical to prevent unnecessary medical or surgical interventions. Tuberculous abscesses are a rare presentation of abdominal TB. Herein, we report an unusual case of an intraperitoneal tuberculous abscess in a 50-year-old male patient who presented with dull abdominal pain in the absence of fever. On computed tomography (CT), the lesion appeared as an isolated para-gastric mass, initially raising suspicion of a tumor. Additionally, we perform a review of relevant literature to improve the understanding of this uncommon form of abdominal TB.

## 2. Case presentation

A 50-year-old man initially presented to a local hospital with a 1-month history of unexplained, dull left upper quadrant abdominal pain on July 6, 2024. The patient denied fever, cough, vomiting, belching, nausea, fatigue, diarrhea, weight loss or night sweats. His medical history was negative for human immunodeficiency virus (HIV) infection, diabetes, immunosuppressive therapy, or previous exposure to TB. A gastroscopy performed at that time revealed chronic nonatrophic gastritis. After taking a course of colloidal bismuth subcitrate as directed by the doctor, his abdominal pain showed no significant improvement.

The patient subsequently presented to the gastroenterology clinic of a tertiary hospital due to persistent symptoms. The records of abdominal physical examination were as follows: flat abdomen; no obstructive abdominal contour or peristaltic wave was observed; soft abdomen; mild tenderness in the left upper abdomen; Murphy sign was negative, and McBurney point was non-tender on palpation.

**Figure 1.**
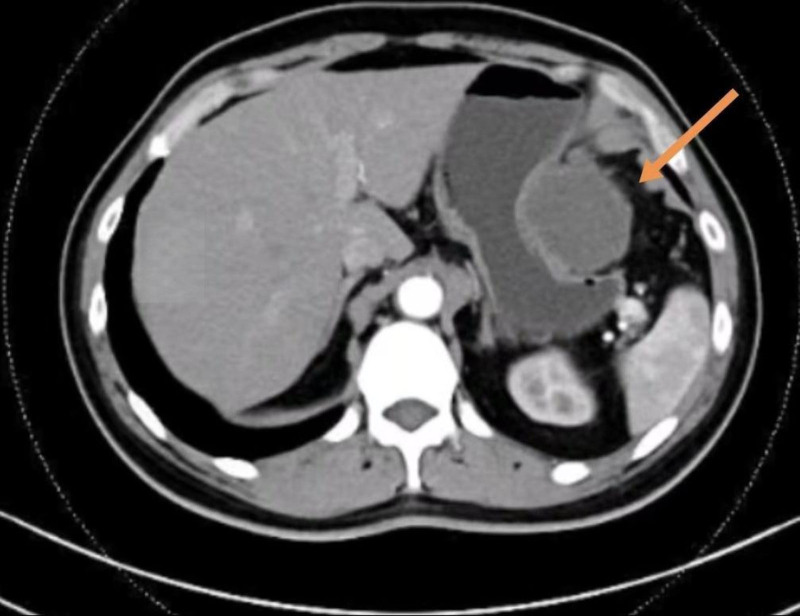
Abdominal contrast-enhanced CT showed a peripherally enhanced, multiseptated hypodense mass abutting the gastric greater curvature. CT = computed tomography.

Abdominal contrast-enhanced CT demonstrated a peripherally enhanced, multiseptated hypodense mass abutting the gastric greater curvature, measuring 68 mm × 55 mm. No significant enlargement of intra-abdominal lymph nodes was noted (Fig. 1).

On July 29, 2024, the patient was admitted to the gastrointestinal surgery department with a suspected diagnosis of gastrointestinal stromal tumor. Initial laboratory evaluation showed elevated level of C-reactive protein at 65.38 mg/L (reference range: 0–10 mg/L). The other laboratory findings including complete blood count, hepatic and kidney function tests, and coagulation profile were within normal limits.

Given the indeterminate nature of the lesion, a diagnostic laparoscopy was planned for direct visualization and tissue sampling. The risks and benefits of the procedure were thoroughly discussed with the patient preoperatively. During laparoscopy, multiple scattered white nodules were identified on the colonic epiploic appendages. Biopsies were obtained, and intraoperative frozen section analysis revealed adipose tissue with no evidence of malignancy.

**Figure 2.**
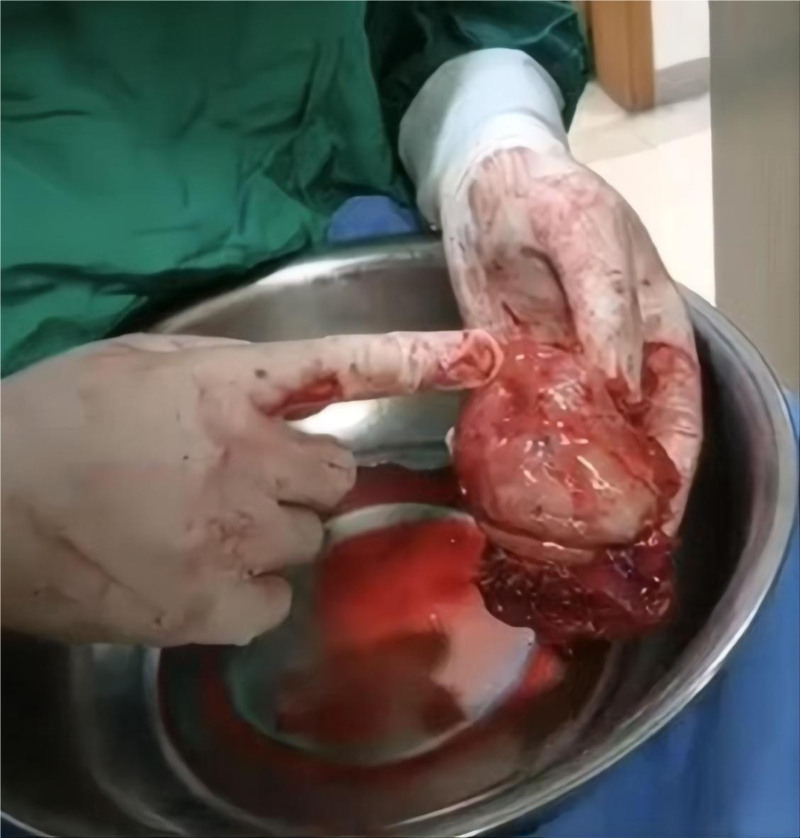
The excised mass with its gastric attachment surface.

**Figure 3.**
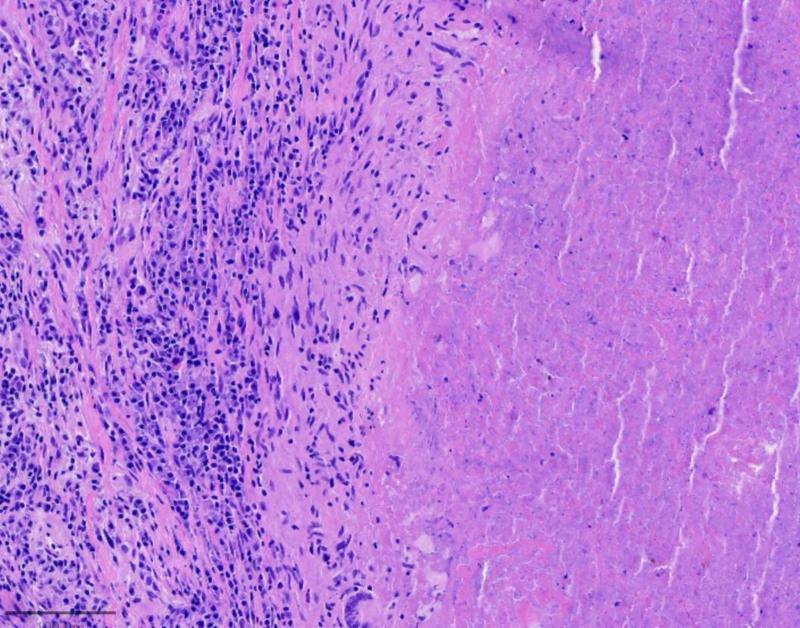
Postoperative pathology showing caseating necrosis and granulomatous inflammation (hematoxylin and eosin, 100 ×).

**Figure 4.**
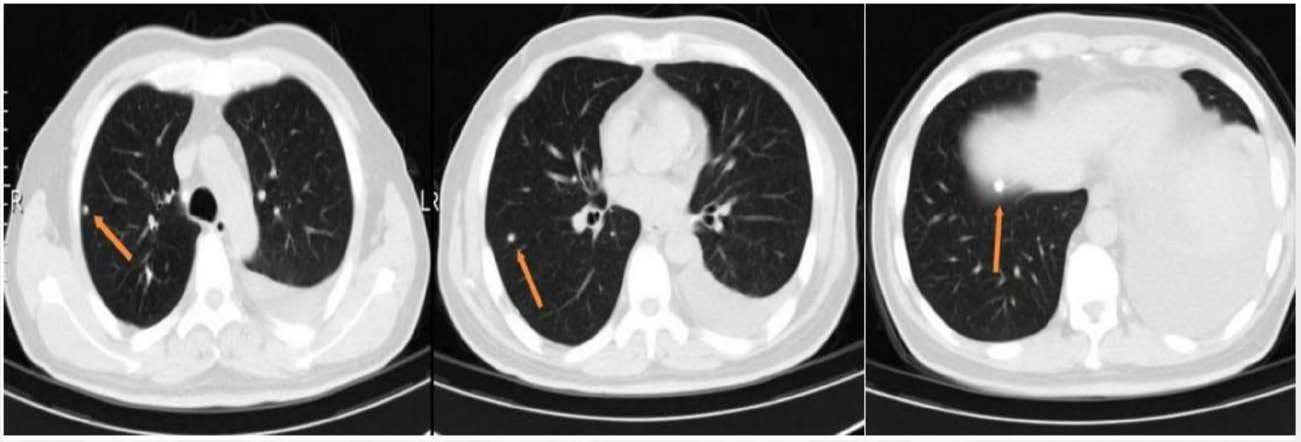
Chest CT showed multiple calcified nodules in the right upper and lower lobes. CT = computed tomography.

Due to inadequate laparoscopic exposure and a nondiagnostic biopsy result, the procedure was converted to an exploratory laparotomy. During laparotomy, a mass measuring approximately 7 cm × 6 cm was identified attached to the gastric fundus, partially adherent to the left diaphragm. The mass was completely excised (Fig. 2). Gastric mucosal exploration showed no involvement. Grossly, the lesion presented as a cystic-solid mass, containing a cavity filled with pus. Histopathological examination of the biopsy specimens suggested caseating necrosis and granulomatous inflammation (Fig. 3). Special stains for acid-fast bacilli (AFB) and fungi (GMS, PAS) were negative. Polymerase chain reaction (PCR) assays detected *Mycobacterium tuberculosis* DNA in the specimens, thereby establishing the diagnosis of tuberculosis. Further diagnostic work-up included chest CT, tuberculin skin test (TST), interferon-gamma release assay (IGRA), sputum examination. TST showed an induration of 22 mm × 18 mm. The result of IGRA was positive. AFB smear and mycobacterial culture of the early-morning sputum specimens were negative. Chest CT demonstrated multiple calcified nodules in the right upper and lower lobes, with a moderate left pleural effusion (Fig. 4). Ultrasound-guided thoracentesis was performed for pleural effusion analysis. Laboratory analysis demonstrated a lymphocyte-predominant exudative effusion, with an adenosine deaminase level of 9.1 IU/L (reference range: 0–25 IU/L). These findings are most consistent with postoperative reactive pleuritis.

With a diagnosis of abdominal TB, the patient was subsequently transferred to a specialized TB center for anti-TB therapy. The regimen consisted of a 2-month intensive phase with isoniazid 300 mg, rifampicin 600 mg, ethambutol 1200 mg, and pyrazinamide 1500 mg daily, followed by a 4-month continuation phase with isoniazid 300 mg and rifampicin 600 mg daily. The patient continued anti-TB therapy at a local TB-designated hospital with an unchanged regimen. He reported stable clinical status with no adverse effects on monthly telephone follow-ups.

## 3. Summary of previous reported cases

Regarding anatomy, intra-abdominal abscesses can be divided into intraperitoneal and visceral abscesses and those located in the retroperitoneal space.^[8]^ In our case, the abscess was located in the peritoneal cavity and did not involve the gastric mucosa. The patient’s final diagnosis was determined as abdominal TB complicating an intraperitoneal tuberculous abscess. We conducted a literature review to summarize the current understanding of this rare condition.

A systematic literature search was conducted in the Pubmed from database inception to May 2025. The search strategy combined Medical Subject Headings (MeSH) and their corresponding entry terms.^[9]^ Terms within the same conceptual group were combined with “OR,” while different concepts were linked with “AND.” The complete search string is provided below:

((“Tuberculosis, Peritoneal”[MeSH] OR “Tuberculosis Peritoneal”[All Fields] OR “Abdominal Tuberculosis”[All Fields] OR “Intraperitoneal Tuberculosis”[All Fields] OR “Intra-abdominal Tuberculosis”[All Fields]) AND (“Abscess”[MeSH] OR “Abscesses”[All Fields]) AND “Case Reports”[ptyp])

OR ((“Tuberculosis”[MeSH] OR “Tuberculous”[All Fields]) AND (“Abdominal Abscess”[MeSH] OR “Abscess Abdominal”[All Fields] OR “Abdominal Abscesses”[All Fields] OR “Abscesses Abdominal”[All Fields] OR “Abscess Intra-Abdominal”[All Fields] OR “Abscesses Intra-Abdominal”[All Fields] OR “Intra-abdominal Abscess”[All Fields] OR “Intra-abdominal Abscesses”[All Fields] OR “Mesenteric Abscess”[All Fields] OR “Subphrenic Abscess”[All Fields])AND “Case Reports”[ptyp]).

The initial search yielded 199 records. After removing 1 duplicate, 198 unique records underwent a 2-stage screening process against predefined eligibility criteria. Inclusion criteria were: case reports or series on human patients; a confirmed diagnosis of intraperitoneal tuberculous abscess (via histopathology, microbiology, or clinical/imaging findings with treatment response). Exclusion criteria were: non-English publications; cases with insufficient diagnostic evidence.

In the first stage, the titles and abstracts of the 198 records were screened, resulting in the selection of 15 articles (13 case reports and 2 imaging-focused case series) for full-text review. In the second stage, the full texts of these articles were assessed. Additionally, we screened the reference lists of the included articles to identify other relevant publications.^[10]^ Following this process, we excluded 3 non-English articles and 1 case report lacking confirmatory diagnostic evidence.

Finally, a total of 11 publications (9 case reports and 2 case series) were included. We cited the 2 case series in the Discussion to support the analysis of radiological features. Nine eligible case reports were included for data synthesis.^[11–19]^ Data were meticulously extracted and presented as descriptive statistics in a table (Table 1). We systematically extracted the following data from each included case: first author and publication year, patient demographics, clinical manifestations, imaging findings, diagnostic methods, treatment regimen and clinical outcomes.

**Table 1:** Clinical features and management of previously reported cases.

	Erenel H et al^[11]^	Elhadidi A et al^[12]^	Imrani K et al^[13]^	Cheung HY et al^[14]^	Solhpour A et al^[15]^	Liu WP et al^[16]^	Schneider et al^[17]^	Pandit V et al^[18]^	Goonetilleke A. et al^[19]^
Publication year	2021	2024	2023	2005	2007	2024	2001	2009	2024
Gender	Female	Female	Female	Male	Male	Male	Male	Male	Male
Age	24	34	36	45	21	72	15	42	42
Comorbidities	Not reported	Not reported	Not reported	Not reported	IgA deficiency	Heart valve disease	Not reported	HIV infection	Not reported
Symptoms	Fever, abdominal pain and weight loss for 3 months	Abdominal pain, fever, vomiting and night sweats for 2 mo	Abdominal pain, fever, night sweats and weight loss	Abdominal pain, fever and diarrhea for 1 wk	Abdominal pain, fever, fatigue, weight loss, cough, and night sweats for 6 mo	Abdominal pain with a palpable abdominal mass for 1 mo	Abdominal pain, night sweats, cough and dyspnea for 11 wk	Vomiting for 6 months, abdominal pain, weight loss and fever for 1 mo	Abdominal pain, acid regurgitation and belching for 2 months, weight loss for 1 mo
TB in other sites	Not reported	Pleura	Not reported	Lung	Brain, lung, bone	Not reported	Pleura	Brain, lung	Not reported
Imaging findings of the abscesses	Ultrasonography showed a heterogeneous mass in the lower abdominal cavity; MRI showed a lobulated left lower abdominal mass containing cystic and necrotic components	CT showed the features of pelvic loco-regional inflammatory processes and turbid collections, suggestive of pelvic abscesses and surrounding peritonitis	CT showed a multiseptated, peripherally enhanced, hypodense mass in the right iliac cavity. Ultrasonography showed a hypoechoic mesenteric mass	Not reported	CT showed multiple subphrenic hypodense lesions	CT showed a right lower abdominal mass with scattered multiple nodules; MRI showed an enhanced foci in the right lower abdominal cavity	T2-weighted MRI demonstrated gelatinous intra-abdominal fluid collections; T1-weighted images showed diffuse enhancement of intra-abdominal masses	Ultrasonography showed a hypoechoic heterogeneous mass in the right lumbar region; CT showed a multiseptated peripherally enhanced hypodense mesenteric lesion suggestive of mesenteric abscess.	CT showed an abscess anterior to the diaphragmatic cruses between the liver and stomach
Abdominal Lymph nodes changes	Not reported	Not reported	Not reported	Laparoscopy revealed pus leaking from matted small bowel mesenteric lymph nodes	Not reported	Not reported	Laparoscopy revealed diffuse intraperitoneal lymph node enlargements	Not reported	CT showed multiple necrotic lymph nodes in the porta hepatis and portocaval region
Peritoneum/mesentery/omentum changes	Not reported	Laparoscopy revealed severe bowel adhesion with miliary peritoneal and abdominal wall nodules	Not reported	Laparoscopy revealed a large amount of purulent peritoneal fluid	Not reported	CT suggested scattered multiple nodules in the abdominal cavity	Laparoscopy revealed nodular thickening of the greater omentum	CT suggested omental thickening; Ultrasonography showed thickened bowel loops and mesentry with interloop ascites	Not reported
Biopsy methods	Fine needle aspiration biopsy	Laparoscopic biopsy	Laparoscopic biopsy	Laparoscopic biopsy	Percutaneous abscess drainage	Laparoscopic biopsy	Laparoscopic biopsy	Fine needle aspiration biopsy	Fine needle aspiration biopsy
Diagnostic methods	PCR of the aspirates was positive for MTB	Histology of the biopsy specimens showed necrotizing epithelioid granulomatous inflammations with the presence of AFB	Histo-bacteriological study confirmed the origin of tuberculosis	Histology of the biopsy specimens showed necrotizing granulomas with the presence of AFB	Culture of the abscess fluid was positive for MTB	Histology of the biopsy specimens suggested peripheral fibrotic tissue proliferation combined with multinucleated giant cell reaction	Z-N staining of the biopsy specimens showed AFB	Acid-fast staining of the pus sample was positive; PCR of the pus sample was positive for MTB.	PCR of the aspirates was positive for MTB
Therapy	Anti-TB therapy(2HRE/7HR)	Surgical drainage; anti-TB therapy	Surgical drainage; anti-TB therapy	Surgical drainage; anti-TB therapy	CT-guided percutaneous drainage; anti-TB therapy (1SHREZ/3HREZ/8HRE)	Surgical drainage; anti-TB therapy	Anti-TB therapy (HRZ)	Anti-TB therapy	Anti-TB therapy (2HRE/4HR)
Outcome	Clinical improvement	Clinical improvement	Clinical improvement	Clinical improvement	Follow-up CT showed that the subphrenic abscesses shrank	Clinical improvement	Clinical improvement	Clinical improvement; Follow-up CT showed resolution of the abscess	Follow-up ultrasonograph showed resolution of the abscess

AFB = acid-fast bacteria, CT = computed tomography, E = ethambutol, H = isoniazid, MRI = magnetic resonance imaging, MTB = mycobacterium tuberculosis, PCR = polymerase chain reaction, R = rifampicin, S = streptomycin, Z = pyrazinamide.

The 9 cases (6 males and 3 females) ranged in age from 15 to 72 years. Two cases had concurrent immunodeficiency (HIV infection and IgA deficiency respectively). The main symptoms included abdominal pain (9/9), fever (6/9), weight loss (5/9), night sweats (4/9). Five patients had TB involvement in other sites (including pleura, lung, bone, brain). Definitive diagnosis was established through laparoscopic biopsy in 5 patients and image-guided aspiration in 4 patients. All 9 patients had favorable clinical and/or imaging responses to anti-TB therapy. Collectedly, abdominal pain is a universal presenting symptom of this condition. Definitive diagnosis requires histological confirmation via biopsy, and the prognosis with anti-TB treatment is favorable.

## 4. Discussion

Although abdominal TB can develop at any age, it is most common in young and middle-aged people.^[20]^ Among the 9 cases reviewed, the majority (8/9) were under the age of 50. Our case review also identified 2 patients with immunodeficiency disorders who developed multi-organ abscesses.^[15,18]^ Previous study has proposed that the severe pathology associated with HIV/MTB coinfection is caused by a functional disruption of the local immune response within the granuloma. These disruptions decrease the ability of the granuloma to contain MTB, thereby promoting mycobacterial dissemination.^[21]^

The infection routes of abdominal TB are as follows: hematogenous or lymphatic spread; direct spread from infected adjacent lesions; ingestion of infected milk or swallowing sputum with MTB.^[2,22]^ In our case, the patient had no history of drinking unpasteurized milk. There was no evidence of TB involvement in the adjacent abdominal organs. The patient’s chest CT showed multiple calcified nodules in the right upper and lower lobes. We speculated that MTB initially invaded the lung and then spread to the peritoneum via the hematogenous route.

An intraperitoneal tuberculous abscess is a rare and serious form of abdominal TB. Dong et al divided intraperitoneal tuberculous abscesses into 2 types: the “encapsulation type and the “lymph node fusion type.”^[23]^ The “encapsulation type” is formed by the intestinal loops, peritoneum, or mesentery encapsulating the caseous substances. This type of abscess may be associated with fibrotic (dry-plastic) tuberculous peritonitis, which is characterized by peritoneal fibrous adhesions.^[24]^ Elhadidi et al reported a case of abdominal TB, in which laparoscopy revealed severe peritoneal adhesions encapsulating an abscess.^[12]^

According to Dong et al., the “lymph node fusion type” is caused by the fusion of necrotic lymph nodes infected with TB. This type of abscess is characterized by multiple enlarged lymph nodes around the main lesion.^[23]^ Abdominal tuberculous lymphadenopathy commonly involves the mesenteric root, celiac, porta hepatis and peripancreatic lymph nodes.^[24]^ Cheung et al reported a case of tuberculous mesenteric abscess, in which exploratory laparotomy revealed pus leaking from the small bowel mesenteric lymphnodes.^[14]^ Schneider et al reported a case of abdominal TB complicated by abscess-like peritonitis, in which laparoscopy revealed diffuse intraperitoneal lymph node enlargements.^[17]^ In our case, the abscess was more likely to be of the “encapsulation type,” as no significant lymph node enlargement was detected adjacent to the abscess.

The clinical presentations of abdominal TB are often nonspecific. Among the 9 cases reviewed, all presented with abdominal pain; four cases additionally exhibited other gastrointestinal symptoms, such as vomiting, acid regurgitation, belching and diarrhea. These symptoms often overlap with other gastrointestinal diseases. In our case, abdominal pain was the the patient’s only obvious symptom, and the gastroscopy result indicated chronic gastritis, masking the existence of an intraperitoneal abscess.

Abscesses caused by TB are typically characterized as cold abscesses. These abscesses are purulent collections lacking the classic signs of acute inflammation, such as redness, heat, and severe pain.^[25]^ Patients often experience fewer systemic symptoms compared to those with pyogenic abscesses. In our case, the lesion appeared as an solitary abscess. The patient had only dull abdominal pain without fever, which aligns with the localized and indolent nature of an cold tuberculous abscess.

Imaging techniques are crucial in the diagnosis of abdominal TB. CT is a fast and simple diagnostic tool that is the preferred method for detecting intraperitoneal lesions. Previous studies reported that intraperitoneal tuberculous abscesses usually presented as isolated or multiple, multiseptated, peripherally enhanced, hypodense masses with regular or irregular shapes.^[13,18,23,26,27]^ CT can also evaluate abdominal lymph nodes, peritoneum and omentum. Peripheral enhancement with central hypodensity is the most frequent pattern of tuberculous lymphadenopathy.^[23,24,26,27]^ The involvement of peritoneum and omentum may present as smooth uniform thickening or nodular thickening.^[16,18,23,24,27]^

Magnetic resonance imaging (MRI) is usually not the first-line examination for abdominal TB, due to the longer scan time, higher cost and lower availability. However, MRI can be used as a complementary or alternative tool because of its superior soft tissue resolution and lack of ionizing radiation.^[28]^ Unnecessary radiation exposure in pregnant women or patients requiring multiple follow-up can be avoided. Erenel et al described the application of MRI for diagnosing a pelvic tuberculous abscess in a pregnant patient.^[11]^ The diagnostic value of MRI for abdominal TB remains to be further explored.

Ultrasound technology is a fast and convenient tool with the advantage of real-time imaging. Sonographically, tuberculous cold abscesses may appear as hypoechoic or complex cystic collections.^[11,13,18,29]^ Ultrasound is usually used in combination with other imaging techniques due to its limitations, such as operator dependency and restricted field of view.

Although imaging plays an important role, the radiological signs are not the gold standard for diagnosis. A solitary intra-abdominal abscess, as in our case, must be distinguished from malignancies. Importantly, a tuberculous cold abscess must be differentiated from a pyogenic abscess caused by common bacteria. Peritoneal thickening or nodularity may also occur in peritoneal carcinomatosis. Enlarged lymph nodes with peripheral enhancement can also be noted in other diseases such as lymphoma, metastatic malignancy, and Whipple disease.

Image-guided aspiration, diagnostic laparoscopy, and laparotomy provide approaches to obtaining histological specimens from intra-abdominal lesions.^[30,31]^ Aspiration biopsy is minimally invasive and cost-effective but limited by sampling errors. Laparotomy offers definitive diagnosis for complex abdominal pathologies but carries higher complication risks. Laparoscopy is a minimally invasive procedure with high diagnostic accuracy. However, its utility is constrained in patients with adhesions or anatomically challenging lesions, potentially requiring conversion to laparotomy.^[7,31]^ Each technique has its advantages and limitations. The selection of biopsy method requires a comprehensive evaluation of the lesion characteristics and the patient’s general condition.

The gold standard for diagnosing tuberculous abscess is the identification of caseous epithelioid granuloma or AFB on histopathological examination. Culture of MTB from the specimens can also confirm the diagnosis.^[30]^ However, the sensitivity of AFB staining is relatively low, and culture requires several weeks. Molecular techniques such as PCR provide a rapid and sensitive method for diagnosing EPTB, particularly in culture-negative or paucibacillary cases.^[32,33]^ In our case, although AFB were not detected on staining, the combination of caseating granulomas and MTB-PCR positivity confirmed the diagnosis of TB.

Treatment of EPTB consists of lesion debridement and standardized anti-TB therapy. Previous studies have described surgical drainage as an effective intervention for managing intraperitoneal tuberculous abscesses.^[12–14,16]^ In our case, the surgical procedure achieved both diagnosis and lesion debridement. However, radical resection in suspected TB cases carries risks, including iatrogenic dissemination of TB and the formation of enteric fistulae. Surgery in abdominal TB should primarily be performed to target complications or obtain tissue sampling.^[7,30,31]^ Surgeons should include TB in the differential diagnosis of intra-abdominal masses to avoid unnecessary radical resections.

Early initiation of standardized anti-TB therapy after diagnosis is essential to ensure treatment success. Without treatment, the death rate from TB disease is high (about 50%). With treatments recommended by WHO, about 85% of people with TB can be cured.^[34]^ WHO recommends a 6-month drug course for anti-TB therapy. A Cochrane systematic review found no significant difference in clinical cure rates between 6-month and 9-month anti-TB regimens for abdominal TB.^[35]^ However, tuberculous cold abscesses are commonly encapsulated by fibrous tissue,^[26]^ which may limit drug penetration into the lesion core. The clinical benefits of prolonging anti-TB therapy for patients with complex abdominal TB (including cases with intestinal strictures, fistulae, or abscesses) remain to be substantiated by well-designed clinical trials.

Our case highlights the diagnostic challenge of an isolated intraperitoneal tuberculous abscess. It is particularly instructive as the patient presented with localized abdominal pain without systemic symptoms. Furthermore, the CT appearance closely mimicked a malignant tumor. Notably, our case demonstrates the vital role of a multimodal diagnostic strategy (imaging, histopathology and molecular biology techniques).

## 5. Conclusion

Abdominal TB can present with varied clinical manifestations. Intraperitoneal tuberculous abscess is a rare but serious form of abdominal TB. Diagnosis relies on a multimodal diagnostic approach consisting of clinical manifestations, radiographic, histopathological and microbiological evidence. Standard anti-TB therapy, combined with cautious surgical intervention is the key to cure. Tuberculosis should be included in the differential diagnosis of intraperitoneal masses to avoid misdiagnosis and unnecessary interventions, particularly in high-TB-burden countries. Future studies should focus on validating diagnostic algorithms and optimizing treatment protocols for complex abdominal TB cases.

## 6. Limitations

Our study has several limitations. Firstly, this is a single-center case report, which inherently limits the generalizability of our findings. Secondly, the follow-up data regarding the long-term outcomes of anti-TB therapy were often incomplete in the reviewed cases. Future studies with larger cohorts and longer follow-up are needed to address these limitations.

## Acknowledgments

We sincerely thank the patient for his kind cooperation and consent to publish this case report.

## Author contributions

**Conceptualization:** Wenting Li, Zhikai Zhao, Wei Sha.

**Data curation:** Wenting Li, Zhikai Zhao.

**Formal analysis:** Wenting Li, Zhikai Zhao.

**Investigation:** Wenting Li, Zhikai Zhao.

**Resources:** Wei Sha.

**Writing – original draft:** Wenting Li, Zhikai Zhao.

**Writing – review & editing:** Wei Sha.
